# Analysis of Trends in Screening, Assessment, and Monitoring of Gestational Diabetes Mellitus Through Bibliometric Analysis

**DOI:** 10.1155/jdr/6825254

**Published:** 2026-04-19

**Authors:** Guangzhen Fu, Shuang Hu, Yunhui Qu

**Affiliations:** ^1^ Department of Clinical Laboratory, Key Clinical Laboratory of Henan Province, The First Affiliated Hospital of Zhengzhou University, Zhengzhou, 450001, China, zzu.edu.cn; ^2^ Genetic and Prenatal Diagnosis Center, The First Affiliated Hospital of Zhengzhou University, Zhengzhou, 450001, China, zzu.edu.cn

**Keywords:** assessment, bibliometric analysis, gestational diabetes mellitus, latent Dirichlet allocation, monitoring, screening

## Abstract

**Objective:**

This study aims to explore recent literature on gestational diabetes mellitus (GDM) screening, assessment, and monitoring and identifying research hotspots and future trends.

**Methods:**

In this study, bibliometric methods were employed to analyze the literature related to the screening, assessment, and monitoring of GDM retrieved from the Web of Science Core Collection (WoSCC). Specifically, the analysis focused on the annual publication and citation trends of relevant literature, collaborative networks involving countries, institutions, authors, and journals, keyword co‐occurrence analysis, reference co‐citation analysis, historical evolution of the research field, and topic modeling.

**Results:**

The results show a 50% rise in publications on gestational diabetes over the past 5 years, with the field experiencing distinct developmental stages from 1961 to 2026. China ranks first in global publication volume, while the United States leads in citation impact and international collaboration intensity. Keyword analysis identified three core clusters and a “three jumps and two rises” evolution pattern of citation bursts, with machine learning and adverse pregnancy outcomes emerging as ongoing high‐burst‐strength keywords. Latent Dirichlet allocation (LDA) topic modeling classified 16 optimal topics into four groups: Screening and Diagnostic Approaches, Pathophysiology and Molecular Mechanisms, Environmental, Social and Behavioral Determinants, and Clinical Management, Complications and Health Outcomes.

**Conclusion:**

The focus of GDM screening, assessment, and monitoring is shifting from traditional oral glucose tolerance test (OGTT)‐based diagnosis to biomarker‐based early prediction and AI‐driven digital monitoring throughout pregnancy, highlighting the importance of patient characteristics and risk factors. Future research will contribute to improving clinical practices for gestational diabetes and enhancing maternal and infant health. This study’s integrated bibliometric and LDA topic modeling approach clarifies the knowledge structure and evolutionary trends of the GDM screening‐assessment‐monitoring continuum, providing targeted new perspectives for further exploration in related fields.

## 1. Introduction

Gestational diabetes mellitus (GDM) is a common metabolic disorder during pregnancy, and its incidence has significantly increased globally in recent years, becoming an important public health issue affecting maternal and infant health [1]. GDM not only poses various adverse effects on the health of pregnant women, such as increased risks of hypertensive disorders, cesarean delivery, and postpartum hemorrhage, but also severely threatens the health of fetuses and newborns, leading to conditions such as intrauterine growth restriction, macrosomia, neonatal hypoglycemia, and neonatal respiratory distress syndrome [2]. Furthermore, GDM patients and their offspring face a higher long‐term risk of developing type 2 diabetes mellitus (T2DM) and cardiovascular diseases [3].

The rise in GDM not only brings direct risks of hyperglycemia during pregnancy but also affects the long‐term metabolic and cardiovascular health of mothers and their offspring postpartum. Therefore, early screening, precise assessment, and effective monitoring have become key components of clinical management [4, 5]. With the continuous updating of diagnostic criteria and advancements in testing technologies, research on GDM has shown a complex trend of multidimensional and interdisciplinary development, covering areas from early risk prediction, optimization of screening methods, innovation in metabolic monitoring techniques, to in‐depth exploration of the impact of abnormal glucose metabolism during pregnancy on maternal and infant outcomes [6–8].

In clinical practice and academic research, the “screening, assessment, and monitoring” of GDM constitute the core technical aspects of the management chain [9]. However, this field has long faced significant international discrepancies and methodological evolution. Gestational diabetes screening is fundamental for identifying high‐risk populations and timely interventions [10]. The one‐step 75 g oral glucose tolerance test (OGTT) recommended by the IADPSG coexists with the two‐step method (50 g glucose challenge test (GCT) + 100 g OGTT) supported by organizations like ACOG, reflecting ongoing trade‐offs between sensitivity, specificity, cost, and operability [11]. The one‐step method typically employs the 75 g OGTT conducted between 24 and 28 weeks of gestation [12]; the two‐step method involves an initial 50 g GCT, followed by a 100 g OGTT if the results are abnormal [13]. These guideline differences not only reflect the diversity of epidemiological characteristics across different populations but also reveal that a single screening model may not meet the diverse clinical needs globally. The sensitivity and specificity of different screening methods have their advantages and disadvantages and are influenced by various factors such as ethnicity, region, and dietary habits [14]. Meanwhile, the traditional OGTT, due to its complexity, time delays, and issues with maternal tolerance, has prompted research hotspots to shift towards earlier and more convenient screening tools, including risk prediction models based on serum biomarkers. The assessment of GDM encompasses a comprehensive consideration of various aspects, including the pregnant woman’s blood glucose levels, degree of insulin resistance, risk of complications, and fetal growth and development. Accurate assessment aids in formulating individualized treatment plans, including dietary management, exercise guidance, and pharmacotherapy, to control blood glucose levels and reduce maternal and infant complications. Additionally, monitoring of GDM patients is also an important means to ensure treatment effectiveness and the safety of both mother and baby. Blood glucose monitoring is central to GDM management, including self‐monitoring of blood glucose (SMBG) and glycated hemoglobin (HbA1c) testing. Regular monitoring of the pregnant woman’s blood pressure, weight, and fetal development is also necessary to timely adjust treatment plans [15].

In recent years, with continuous advancements in medical technology and a deeper understanding of GDM, research on GDM screening, assessment, and monitoring has been increasing. However, these studies exhibit significant heterogeneity in research design, sample size, characteristics of study subjects, and research outcomes, leading to some controversies and uncertainties in clinical management strategies for GDM. Bibliometric analysis, a scientific method for analyzing literature, can quantitatively analyze and visually present the literature output, research hotspots, and trends in a specific research field, providing valuable reference information for researchers and clinicians [16]. Although existing bibliometric studies have provided a panoramic view of the overall field of GDM [8], there is still a lack of in‐depth mapping analysis specifically targeting the evolution, knowledge structure, and future trends of the “screening, assessment, and monitoring” technical aspects, particularly in revealing the specific shifts in technical focus hidden within the vast literature using techniques such as topic modeling. This study aims to conduct a systematic bibliometric analysis, combined with latent Dirichlet allocation (LDA) topic modeling, focusing on the field of GDM screening, assessment, and monitoring, with the objectives to: (1) quantify the research output and collaboration network in this field; (2) identify the historical evolution and current frontiers of research hotspots; (3) reveal how academia is transitioning from a “diagnosis‐oriented” approach to “predictive warning” and “comprehensive monitoring” against the backdrop of controversies in diagnostic standards and technological innovations. By filling the knowledge gap in this specific field, this study aims to provide empirical evidence and strategic perspectives for unifying clinical practice, optimizing resource allocation, and guiding future technological innovations.

## 2. Materials and Methods

### 2.1. Data Sources and Retrieval Strategy

There are multiple biomedical databases in bibliometric research, including Web of Science (WOS), Scopus, PubMed, MEDLINE, and Embase. Although integrating different databases can provide more information, there is a significant amount of duplicate data among them. Combining data from different sources may introduce unnecessary confounding factors, affecting data quality and ultimately impacting experimental conclusions. Choosing a single, high‐quality, authoritative database as the data source is a common strategy for bibliometric analysis, and Web of Science Core Collection (WoSCC) was selected as the data source for this informatics analysis for multiple core research demands of bibliometric analysis. First, it minimizes confounding bias caused by duplicate data from multiple databases and ensures the uniformity and accuracy of the research data. Second, WoSCC has comprehensive coverage of high‐impact journals in the biomedicine and public health fields, which ensures the inclusion of core influential studies in the field of GDM screening, assessment, and monitoring. Third, its standardized metadata structure and reliable citation association system provide a solid foundation for analyzing collaborative networks (at the national, institutional, and author levels) and citation trends, which are the core research objectives of this study. These advantages are consistent with the industry consensus that WoSCC is the gold standard for citation‐based bibliometric analysis.

**Figure 1:**
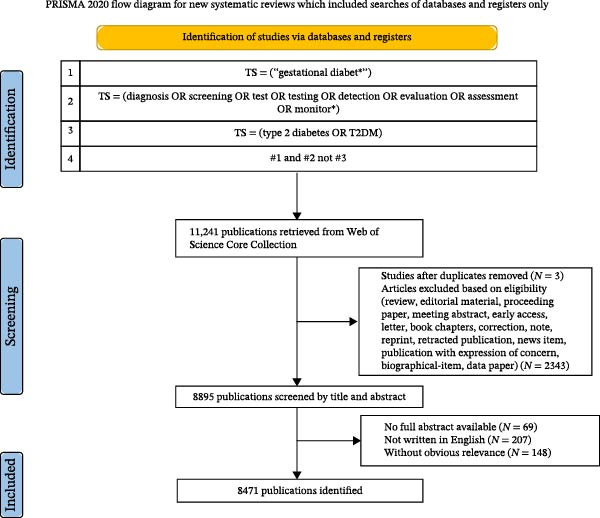
Search strategy and analysis flowchart for gestational diabetes mellitus screening.

We acknowledge the limitations of a single database selection and clarify the potential impact of excluding other major biomedical databases on the research coverage of the GDM field. For the Scopus database, which covers a larger number of journals (especially regional journals in Europe and Asia), the exclusion may lead to insufficient representation of some clinical studies in non‐English‐speaking regions. However, existing studies have confirmed that the overlap rate of WoSCC and Scopus in core biomedical journals is about 85%, and the two are consistent in the identification of high‐impact research trends, so the exclusion of Scopus will not change the core research hotspots and long‐term trends identified in this study. For the PubMed/Medline database, a large number of clinical practice guidelines, case reports, and low‐impact factor specialty journals not included in WoSCC are included. These excluded literatures tend to focus on themes such as the clinical implementation of GDM screening guidelines and the optimization of regional screening protocols, which may lead to an underestimation of the research volume of such themes in this study, because such practice‐oriented studies are more likely to be published in obstetrics and gynecology specialty journals indexed by PubMed rather than interdisciplinary journals indexed by WoS. The data source for bibliometric analysis is the WoSCC. The retrieval date was October 21, 2025. The following search strategy was used: TS = (“gestational diabet ^∗^”) AND TS = (diagnosis OR screening OR test OR testing OR detection OR evaluation OR assessment OR monitor ^∗^) NOT TS = (type 2 diabetes OR T2DM). A total of 11,241 articles were obtained, with the literature type limited to “article” and other types excluded; language restrictions were set to English. Data were manually screened to ensure the authenticity and reliability of the research data, with two researchers in the field independently conducting the data screening. In cases of disagreement, a third researcher participated in the discussion. After manual reference screening, a total of 8471 documents were obtained. The retrieval and download of data were conducted on the same day to minimize confounding bias caused by daily database updates. Complete records were downloaded in formats including plain text, BibTex, tab‐delimited files, and Excel for further analysis. The detailed retrieval and screening processes are shown in Figure 1.

### 2.2. Data Analysis, Visualization, and Topic Modeling

#### 2.2.1. Data Analysis and Visualization

In this study, we imported the retrieved data into Citespace (version 6.4.R1 Advanced), VOS viewer (version 1.6.20), R (version 4.4.1) with the bibliometrix package, KH Coder (3b07d), an online word cloud generator (https://wordart.com/), and an online chart‐making website (https://mm.edrawsoft.cn/app/create) to analyze the published research and create visual maps.

VOS viewer can perform co‐authorship analysis, co‐occurrence analysis, and co‐citation analysis on large‐scale literature data, visualizing knowledge maps that represent authors, journals, and other relevant information. It has been widely used in bibliometric analysis research [17, 18]. Different nodes represent authors, countries, institutions, journals, and keywords. The size of the nodes indicates the corresponding citation or citation count. Links between nodes represent collaboration and co‐occurrence relationships. The colors of the nodes and lines represent different clusters or corresponding years or average references.

CiteSpace is another software focused on visualizing and analyzing scientific literature networks, helping to explore knowledge domains and identify important research trends. Its functions include visualizing co‐citation networks, revealing significant literature and influential authors in the field, and detecting emerging trends through analysis of co‐citation bursts. Additionally, CiteSpace can visualize keyword co‐occurrence networks, helping to identify thematic clusters and their evolution over time. We used CiteSpace (6.4.R1) for keyword and reference clustering analysis and temporal distribution to determine the development dynamics and future trends in the research field [19]. The bibliometrix package in R created a historiograph of references, showcasing the citation relationships among the literature.

#### 2.2.2. LDA Topic Modeling

Topic modeling is a natural language processing (NLP) technique used to discover latent themes in published literature. LDA is a popular topic modeling method that effectively handles large amounts of unstructured text [20]. LDA operates by generating a term function vocabulary and analyzing word frequency co‐occurrences in documents. Its core objective is to extract hidden topics from large‐scale complex text information, describe each topic with specific terms, and thus capture key information from the text content. LDA divides text content into three layers: document, topic, and word, and represents each document as a distribution of topics. The mathematical framework of the model is defined as follows: The probability of a word appearing in a document is the product of the probability of the word appearing in a topic and the probability of the topic appearing in the document: P(w | d) = P(w | t) × P(t | d) where w, t, and d represent word, topic, and document, respectively. The joint probability distribution of the model is further expressed as: $\text{p}\left({\text{w}}_{\text{m}},{\text{z}}_{\text{m}},\theta_{\text{m}},\Phi |\alpha ,\beta \right)=\Pi_{n=1}^{N_{m}}p\left(\Phi |\beta \right)p\left(\theta_{\text{m}}|\alpha \right)p\left({\text{z}}_{\text{m},\text{n}}|\theta_{\text{m}}\right)p\left({\text{w}}_{\text{m},\text{n}}|\Phi ,{\text{z}}_{\text{m},\text{n}}\right)$ where *N*_m_ denotes the length of document m, and z_m,n_ is the topic generated in document m.

To obtain the final topic results, we adopted the Gibbs sampling method based on Markov Chain Monte Carlo (MCMC) for LDA model estimation, ensuring stable and reliable parameter inference.

##### 2.2.2.1. Core Parameter Settings

Hyperparameters: Symmetric prior distribution was adopted to ensure the rationality of topic distribution, with *α* = 0.1 and *β* = 0.01; Number of iterations: Set to 1000 iterations, with the convergence criterion defined as “perplexity change < 1% for 50 consecutive iterations.” The model finally converged at 850 iterations; Random seed: Fixed at 42 to ensure the reproducibility of modeling results; Number of topics: Determined as 16 through multiple classic LDA topic quality evaluation indicators (Griffiths2004, CaoJuan2009, Arun2010, Deveaud2014) [21–23] (see Figure S1 for details).

##### 2.2.2.2. Optimal Topic Number Determination

In Figure S1, the optimal number of topics was determined to be 16 in this study using four classic evaluation metrics for LDA topic quality: Griffiths2004, CaoJuan2009, Arun2010, and Deveaud2014. The rationale is as follows: First, smaller values of the three metrics—Griffiths2004, CaoJuan2009, and Arun2010—indicate better topic discrimination and stability. As shown in the figure, the values of these three metrics decrease continuously with an increasing number of topics, but the downward trend slows significantly near 16 topics. This suggests that the discrimination between topics is sufficiently clear at 16 topics, and further increasing the number of topics provides little gain in improving discrimination. Second, a higher value of the Deveaud2014 metric indicates better semantic coherence of topics. The figure shows that this metric reaches a high level and plateaus when the number of topics increases to 16, demonstrating that the internal semantic relevance of each topic is sufficiently strong at 16 topics. Therefore, 16 topics were selected as the optimal number.

##### 2.2.2.3. Topic Labeling Criteria

To ensure the objectivity and reliability of topic labeling, a standardized four‐step process was adopted: Extract the top 20 keywords with the highest probability for each topic (reflecting the core semantic features of the topic); Screen 10 representative documents for each topic (documents with the highest topic membership degree, that is, the strongest correlation with the topic); Synthesize the keyword co‐occurrence patterns and the research objectives, methods, and conclusions of representative documents to refine topic labels; Cross‐validation of labels was conducted by two independent researchers. Disagreements were resolved through discussions with a third expert in the field of GDM research.

For the topic cluster “Environmental, Social and Behavioral Determinants,” the labeling was supported by both dominant keywords and representative articles: the top 20 keywords of the included topics (e.g., “diet,” “lifestyle,” “pollutant,” “health literacy,” “social support”) form a clear semantic cluster around external influencing factors; representative documents mainly focus on the impact of dietary patterns, environmental exposure, and health behaviors on GDM risk, as well as the regulatory role of social factors (e.g., medical accessibility) in GDM management. Thus, the label accurately reflects the core content of the topic cluster and is not overly broad.

The raw data downloaded from WoSCC were first imported into Microsoft Excel 2021 for preliminary organization. We retained only the titles and abstracts of each article to form the original corpus. To ensure the reliability and validity of the results, we established a word frequency threshold, removed common stop words, and defined specific stop words. LDA topic modeling and word cloud generation were conducted using KH Coder and the online word cloud generator. In the final stage, we manually named each topic based on the 10 most prominent articles and 20 thematic terms under each topic.

## 3. Results

### 3.1. Trends in Annual Publications and Citations

**Figure 2:**
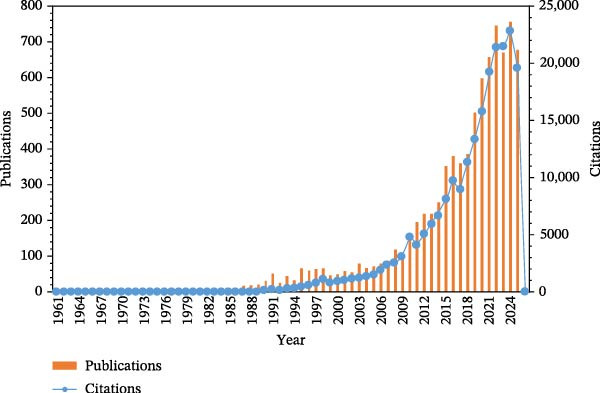
Annual publication output and citation trends.

According to the WOS citation report, as shown in Figure 2, from 1961 to 2026, the overall publication and citation volumes in this field exhibit a clear growth trend, although significant differences exist in growth characteristics across different time periods.

The period from 1961 to 1976 was a nascent phase, with both publication and citation volumes at relatively low levels and fluctuating significantly. The period from 1977 to 1990 marked a growth phase, during which publication and citation volumes began to increase steadily from 1977. The period from 1991 to 2000 was characterized by rapid development, entering the 1990 s when publication and citation volumes entered a phase of rapid growth. The period from 2001 to 2010 was a period of sustained expansion, during which publication and citation volumes continued to grow. The period from 2011 to 2026 represents a maturation and adjustment phase, where, after entering the second decade of the 21st century, publication and citation volumes in this field reached a mature stage but also exhibited fluctuations. In 2011, the publication volume was 196 articles, with 4124 citations; although there were fluctuations in publication and citation volumes thereafter, they remained at a relatively high level overall. In 2021, the citation volume reached 19,191, marking a peak. The 2025 data is relatively low due to only partial data being available at the time of retrieval, and three 2026 articles are present due to early database inclusion by some journals.

Overall, the publication and citation volumes in this field have undergone a process of growth from nascent to rapid development over the past 60 years.

### 3.2. Analysis of National, Institutional, Author, and Journal Collaborations

Based on the results of VOSviewer analysis, this study conducted a comprehensive assessment of bibliometric analysis in the field of “gestational diabetes screening” based on publication volume, citation volume, and average citation counts from various countries. As shown in Figure 3A and Table 1.

Figure 3Collaborative network analysis of countries (A), institutions (B), authors (C), and journals (D).

**Figure:**
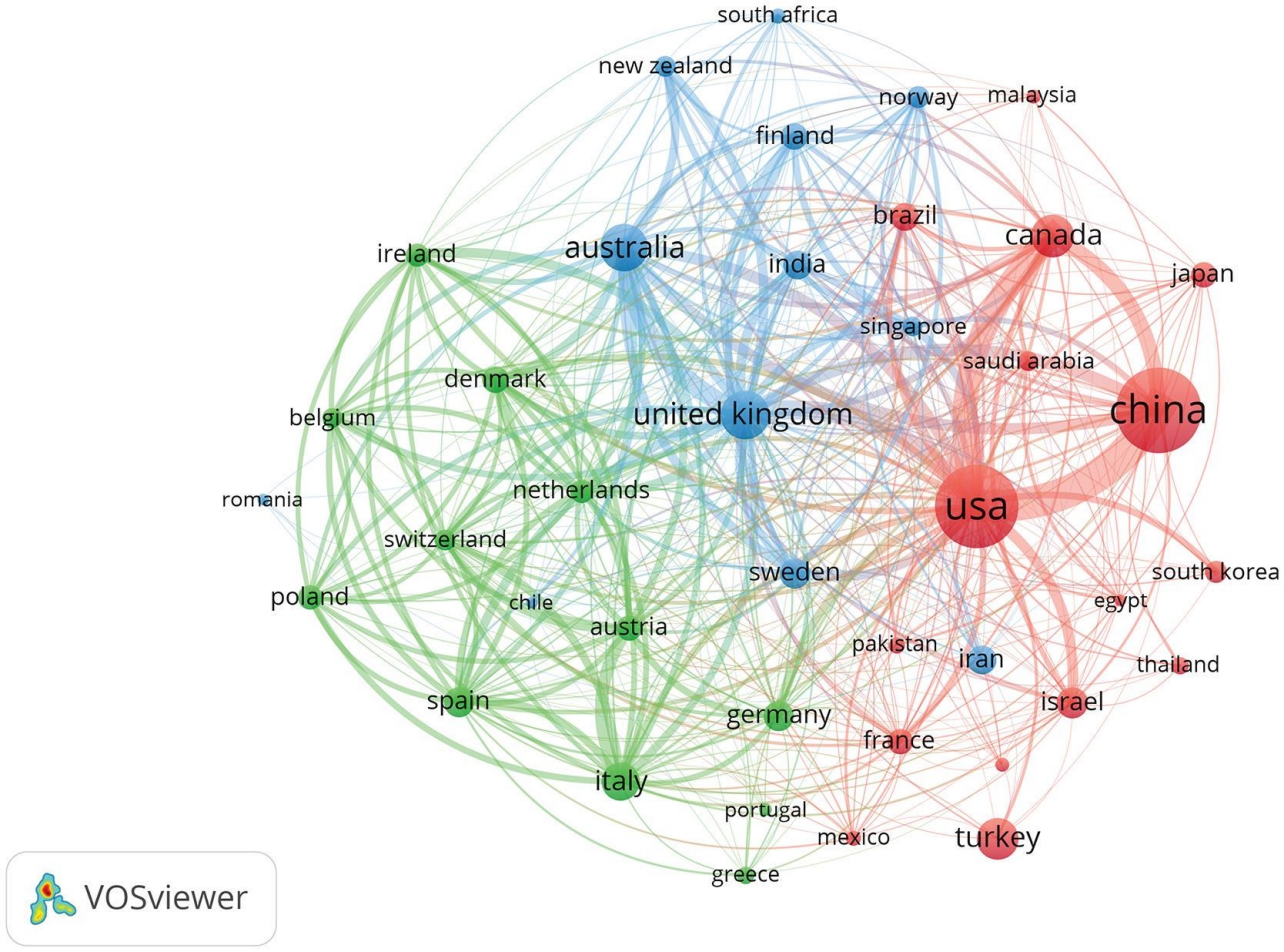
(A)

**Figure:**
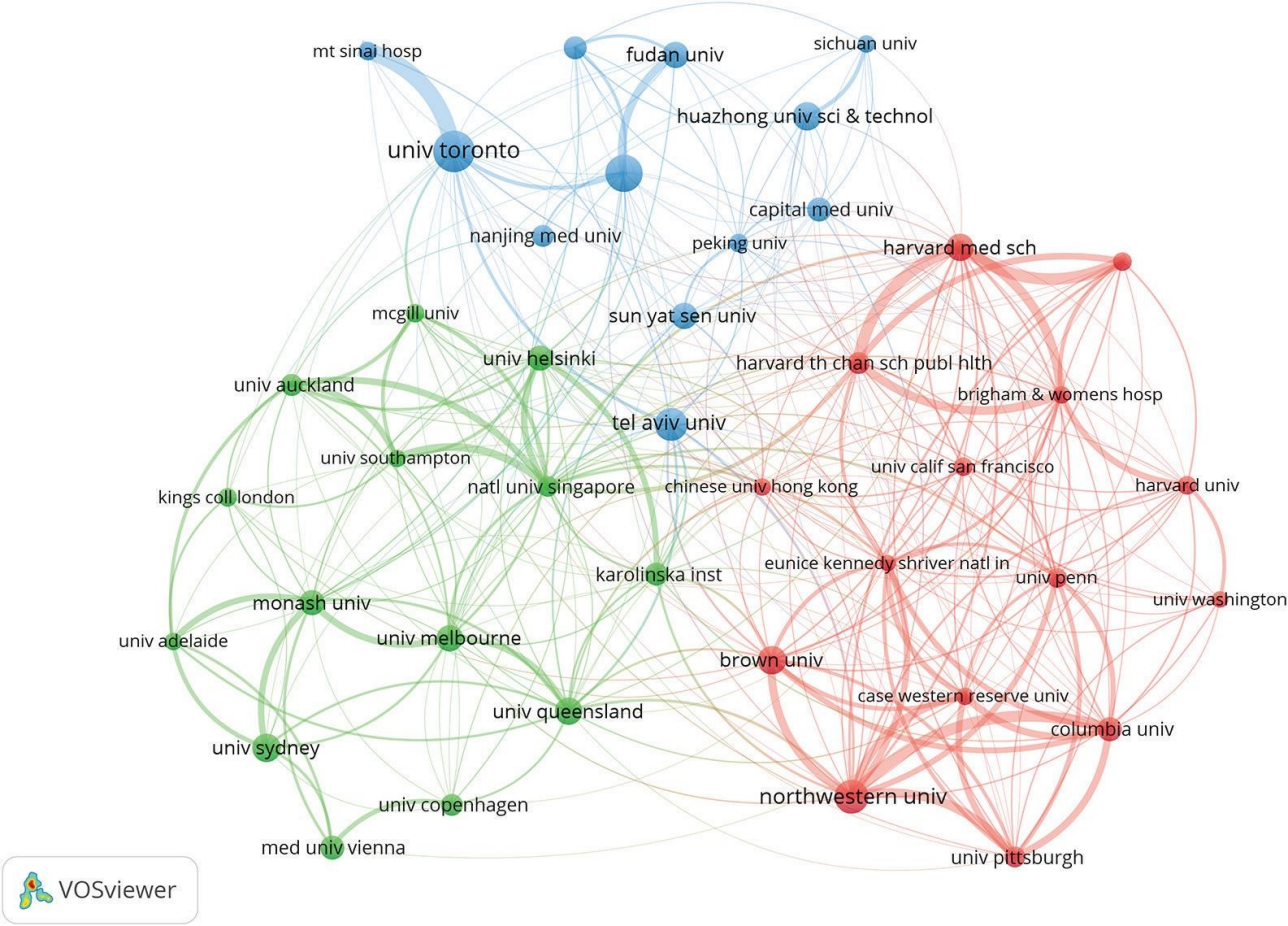
(B)

**Figure:**
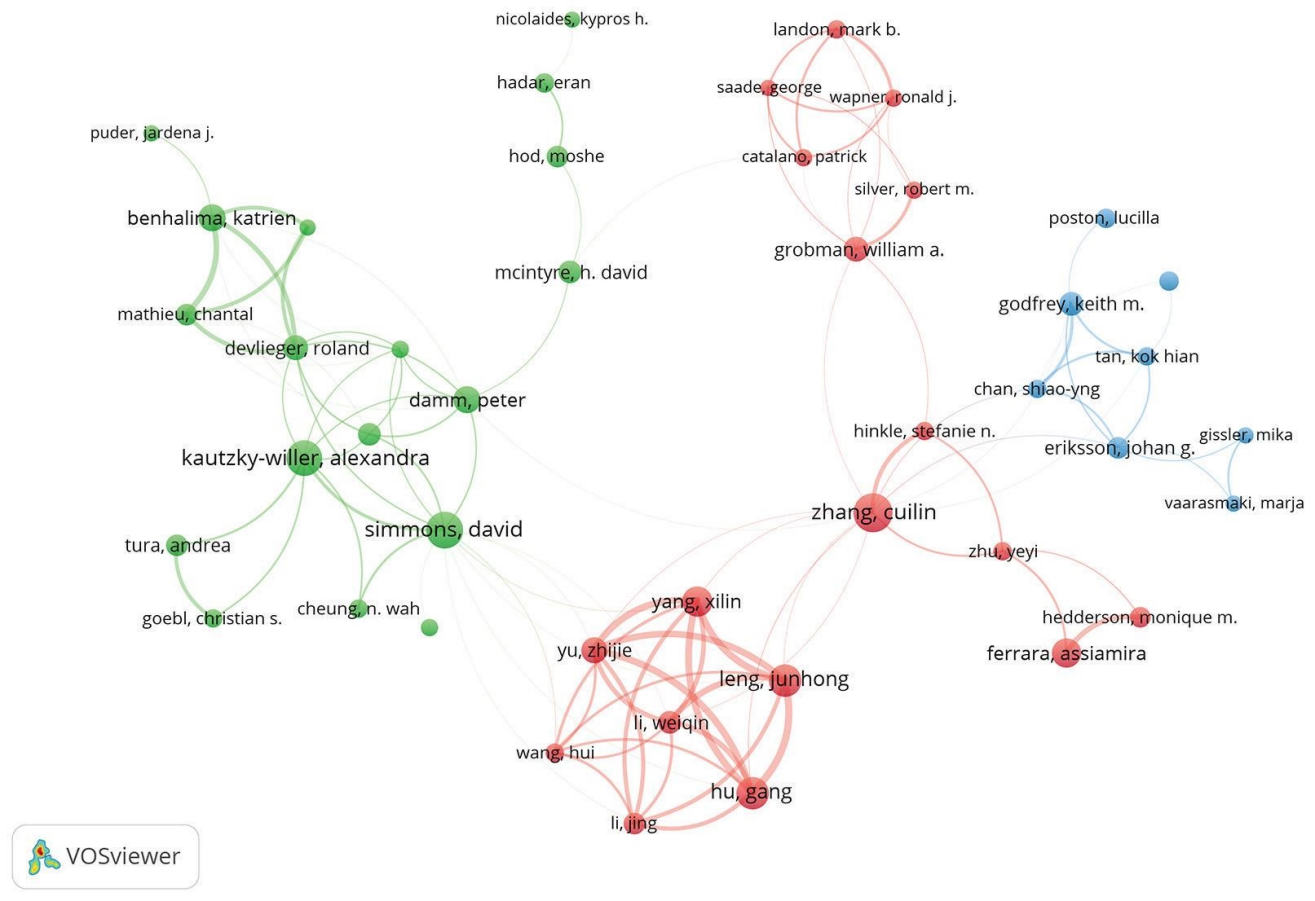
(C)

**Figure:**
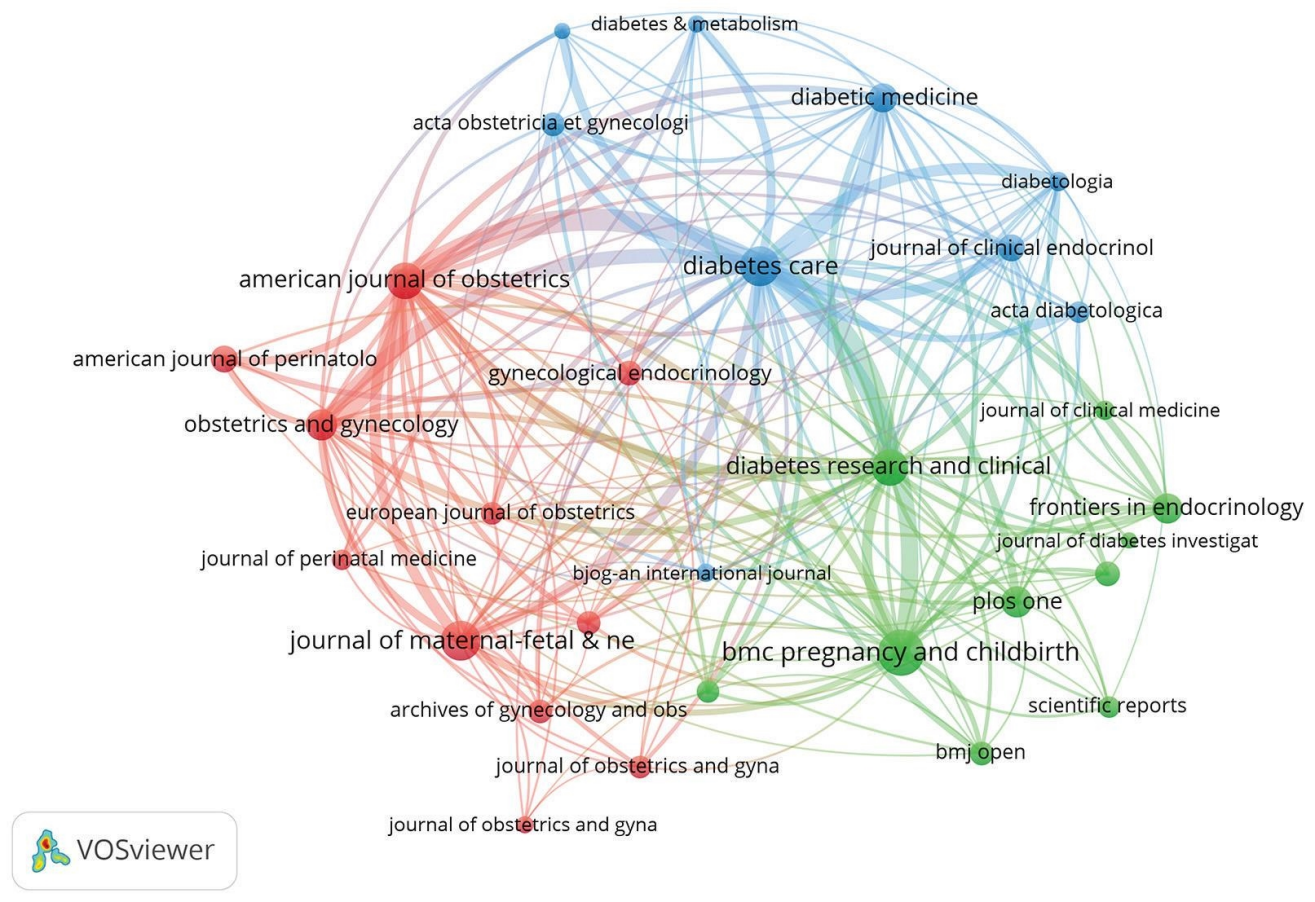
(D)

**Table 1:** Total publications, citation counts, and average citations of countries, institutions, authors, and journals.

Ranking		NP	NC	AC
Country				
1	China	1966	32,312	16.44
2	USA	1875	80,295	42.82
3	United Kingdom	592	24,448	41.30
4	Australia	576	24,757	42.98
5	Canada	461	14,291	31.00
6	Turkey	438	4989	11.39
7	Italy	361	9942	27.54
8	Israel	235	10,859	46.21
9	Spain	228	7275	31.91
10	Germany	217	6126	28.23
Institutions				
1	Univ Toronto	145	5113	35.26
2	Shanghai Jiao Tong Univ	132	2139	16.20
3	Northwestern Univ	120	12,198	101.65
4	Tel Aviv Univ	115	8353	72.63
5	Brown Univ	102	11,255	110.34
6	Univ Sydney	100	2924	29.24
7	Huazhong Univ Sci & Technol	99	1544	15.60
8	Harvard Med Sch	98	2518	25.69
9	Univ Queensland	97	8036	82.85
10	Univ Melbourne	92	2863	31.12
Authors				
1	Zhang, Cuilin	44	1380	31.36
2	Simmons, David	41	1698	41.41
3	Kautzky‐Willer, Alexandra	40	937	23.43
4	Hu, Gang	37	852	23.03
5	Leng, Junhong	37	742	20.05
6	Yang, Xilin	34	1801	52.97
7	Ferrara, Assiamira	33	1827	55.36
8	Damm, Peter	30	1063	35.43
9	Benhalima, Katrien	30	553	18.43
10	Yu, Zhijie	29	697	24.03
Journals				
1	Bmc pregnancy And childbirth	333	5692	17.09
2	Journal of maternal‐fetal & neonatal medicine	262	3754	14.33
3	Diabetes care	259	20,736	80.06
4	American journal Of obstetrics And gynecology	224	15,673	69.97
5	Diabetes research and clinical practice	220	6254	28.43
6	Obstetrics and gynecology	163	9873	60.57
7	Plos one	161	3712	23.06
8	Frontiers in endocrinology	152	1296	8.53
9	Diabetic medicine	145	4737	32.67
10	Journal of clinical endocrinology & metabolism	125	4534	36.27

Abbreviations: AC, average citations (NC/NP); NC, number of citations; NP, number of publications.

In the bibliometric analysis of literature from various countries, China ranks first globally with a publication volume of 1966 articles and a total citation count of 32,312. The United States follows with a publication volume of 1875 articles and a total citation count of 80,295, with an average citation count of 42.82. The United Kingdom ranks third with a publication volume of 592 articles and a total citation count of 24,448, with an average citation count of 41.30.

In the bibliometric analysis of international collaboration, the United States occupies the central position in the global collaboration network. The collaboration intensity between China and the USA is 166, and the collaboration intensity between Canada and the USA is 117, representing the two highest cross‐country collaboration intensities in this field.

According to the results of VOSviewer analysis, the three institutions with the highest publication volumes in this field are the University of Toronto (145 articles, total citations 5113, average citations 35.26), Shanghai Jiao Tong University (132 articles, total citations 1239, average citations 16.20), and Northwestern University (120 articles, total citations 12,198, average citations 101.65). The closest institutional collaboration is between the University of Toronto and Mount Sinai Hospital (collaboration intensity 63), followed by Harvard Medical School and Harvard T.H. Chan School of Public Health (collaboration intensity 42).

According to the analysis results from VOS viewer, the three authors with the most publications are Zhang Cuilin (44 publications, total citations 1380, average citations 31.36), Simmons David (41 publications, total citations 1698, average citations 41.41) and Kautzky‐Willer Alexandra (40 publications, total citations 937, average citations 23.43). The strongest author collaboration is between Hu, Gang and Leng, Junhong (collaboration intensity 31); Hu, Gang also has a collaboration intensity of 29 with both Yang, Xilin and Yu, Zhijie.

According to the analysis results from VOS viewer, the three journals with the highest publication volumes are “BMC Pregnancy and Childbirth” (333 publications, total citations 5692, average citations 17.09), “Journal of Maternal‐Fetal & Neonatal Medicine” (262 publications, total citations 3,754, average citations 14.33), “Diabetes Care” (259 publications, total citations 20,736, average citations 80.06). The closest journal collaboration is between “American Journal of Obstetrics and Gynecology” and “Diabetes Care” (collaboration intensity 364), followed by “American Journal of Obstetrics and Gynecology” and “Obstetrics and Gynecology” (collaboration intensity 354).

### 3.3. Keyword Analysis

A bibliometric keyword analysis was conducted on the literature in the field of gestational diabetes mellitus screening. First, the keywords in the original data were standardized: generic terms with no clear research orientation and low‐frequency terms lacking representativeness were excluded, and the singular/plural forms, tenses, and isomers of the keywords were uniformly consolidated. Finally, 61 core keywords were selected. Based on the clustering rate and occurrence frequency of the keywords, they were divided into three main clusters (Figure 4A) and named in accordance with their cluster characteristics. The specific features of each cluster are as follows: Cluster 1 (Red): OGTT‐based Screening, Diagnosis and Pregnancy Outcome Management of Gestational Diabetes Mellitus.

Figure 4Keyword co‐occurrence cluster network analysis based on VOSviewer (A); keyword co‐occurrence temporal network analysis based on VOSviewer (B); CiteSpace keyword cluster timeline view (C); and citation bursts (top 20 keywords) (D).

**Figure:**
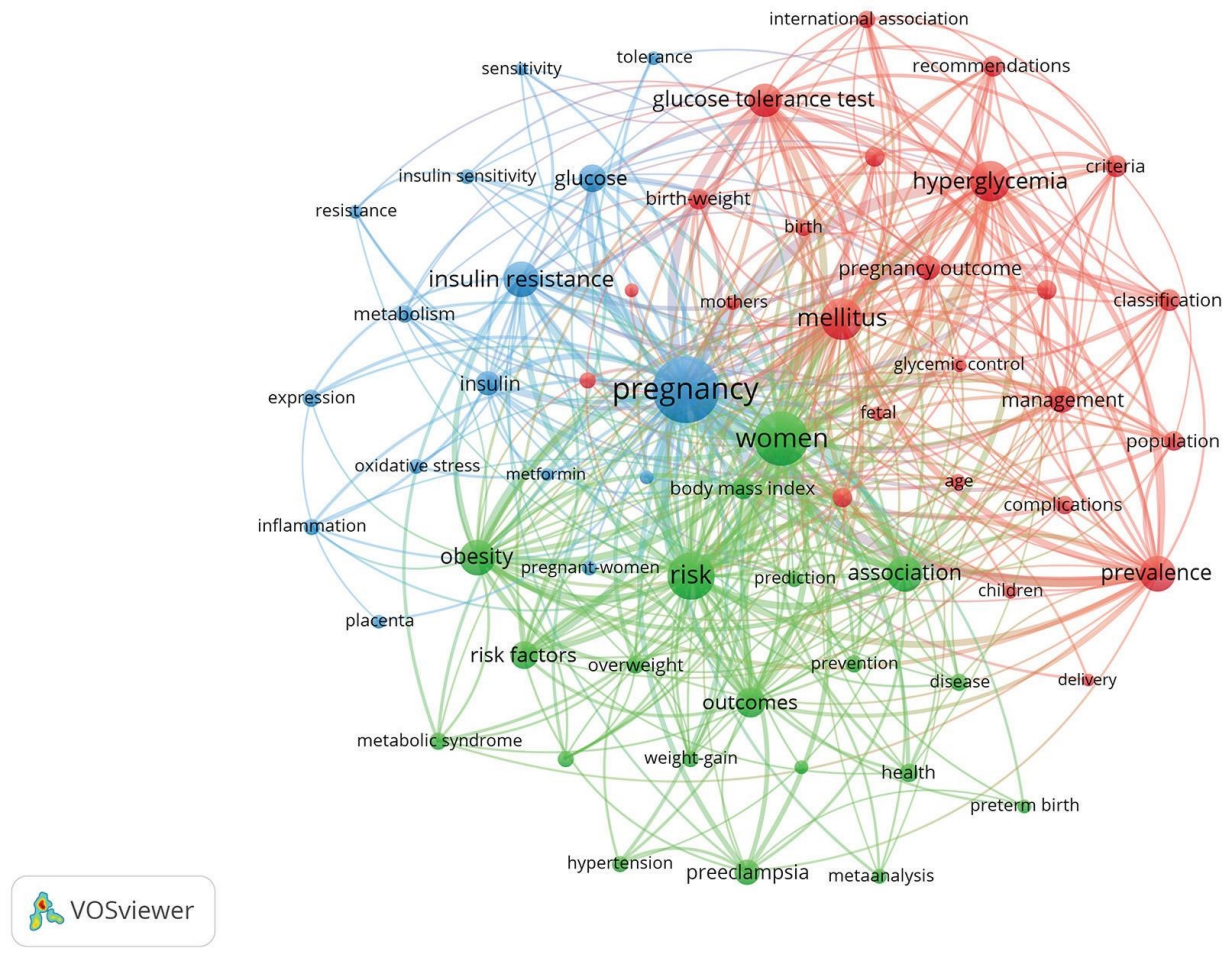
(A)

**Figure:**
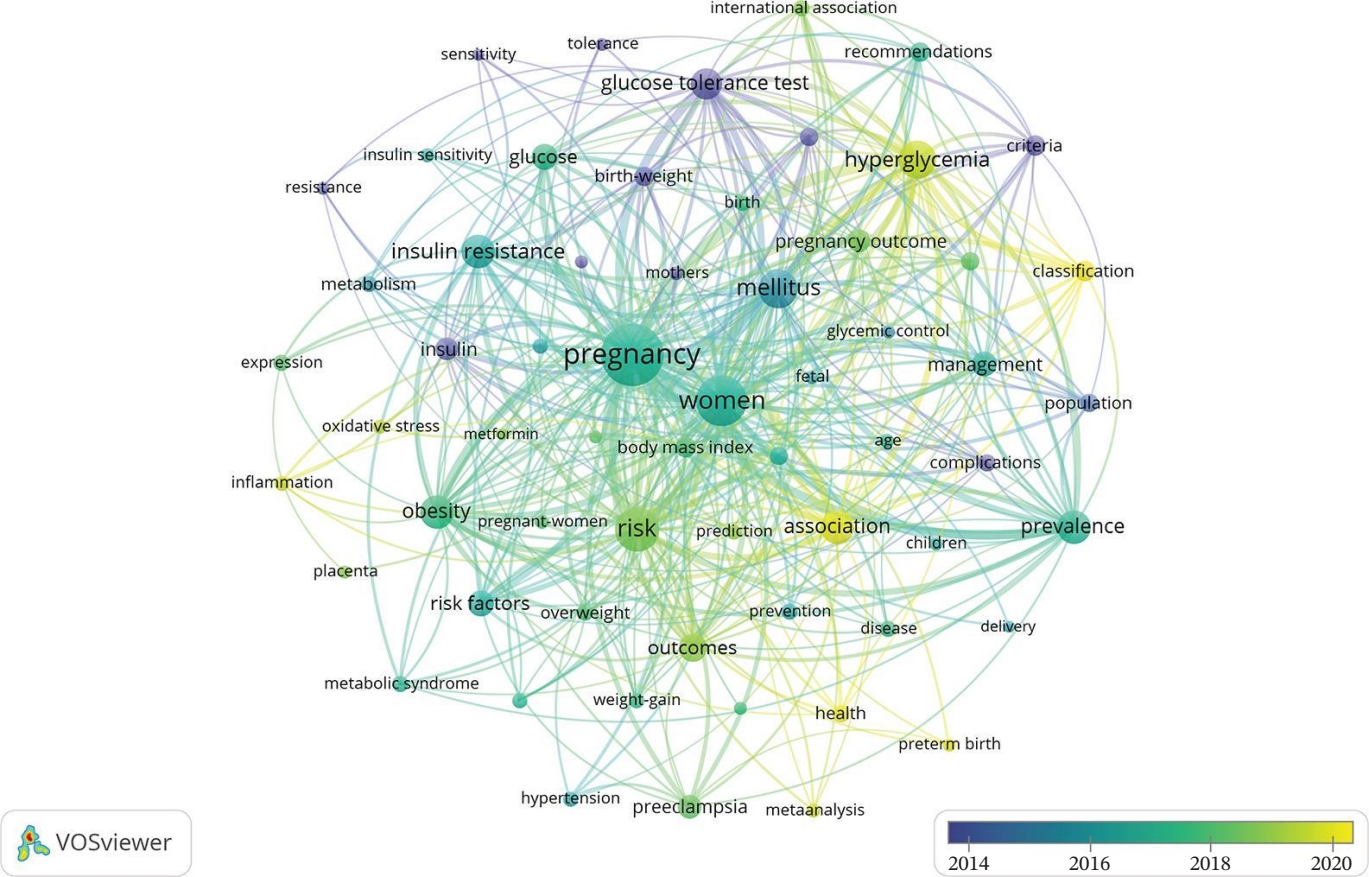
(B)

**Figure:**
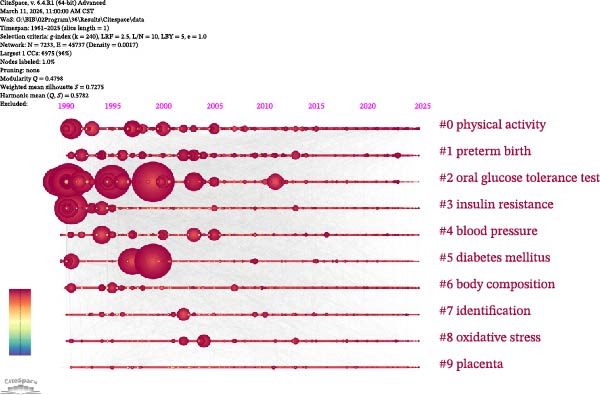
(C)

**Figure:**
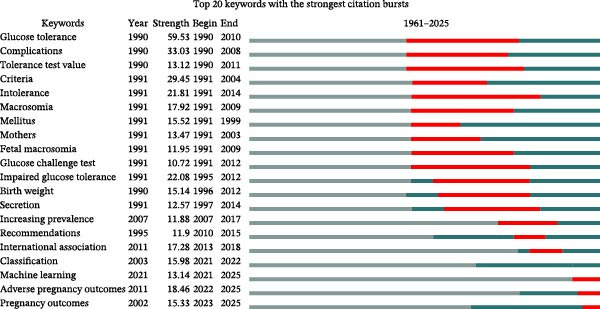
(D)

This cluster takes mellitus (1369), hyperglycemia (1266), prevalence (1024) and glucose tolerance test (891) as the core high‐frequency keywords, and also includes criteria (412), international association (247), management (571), pregnancy outcome (498), complications (287), macrosomia (340), birth‐weight (360), glycemic control (158), and other keywords.

Cluster 2 (Green): Screening of High‐risk Factors and Risk Prediction of Maternal‐fetal Outcomes in Gestational Diabetes Mellitus.

This cluster takes risk (1744), women (2224), obesity (1021), association (1012) and risk factors (611) as the core high‐frequency keywords, and also includes body mass index (399), weight‐gain (223), preeclampsia (507), pregnancy complications (163), preterm birth (178), hypertension (222), prediction (215), prevention (243) and other keywords.

Cluster 3 (Blue): Mechanisms of Insulin Resistance in the Context of Gestational Diabetes Mellitus Screening.

This cluster takes pregnancy (3345) and insulin resistance (1034) as the leading keywords, and also includes glucose (636), insulin (486), metabolism (270), inflammation (233), placenta (159), oxidative stress (196), metformin (150), postpartum (161), and other keywords.

VOS viewer marks the keywords in the figure with different colors based on their average year of occurrence (Figure 4B). The color gradient from deep purple to yellow represents the average occurrence year of keywords, with yellow and light green nodes indicating keywords with a more recent average occurrence year (e.g., hyperglycemia).

The keyword timeline map generated by CiteSpace software (Figure 4C) shows the temporal distribution of research themes: the OGTT is a stable research theme from the 1990s to the 2020s, with node color changing from yellow to deep red and node size peaking from 1990 to 2000 then gradually decreasing.

Citation bursts analysis of keywords (Figure 4) reveals an evolution pattern of “three jumps and two rises”: the first phase: “glucose tolerance” peaks with a burst intensity of 59.53; the second phase: “macrosomia,” “fetal macrosomia,” and “impaired glucose tolerance” reach a peak burst intensity of 22.08; the third phase: “recommendations” (11.9) and “international association” (17.28) burst simultaneously. The “two rises” stage starting in 2021: “machine learning” (burst intensity 13.14, ongoing burst) and “adverse pregnancy outcomes” (burst intensity 18.46, the highest since 2010) show continuous high burst strength. The overall peak burst intensity of keywords shows a gradual decline from 59 to 22–18, while the burst cycle shortens from 20 to 5 years. The emerging themes of “machine learning” and “pregnancy outcomes” are predicted to maintain high burst strength until 2025.

### 3.4. Reference Analysis and Historical Evolution

In this study, we carried out a detailed bibliometric analysis of the literature using CiteSpace software with a time slice of 1 year (slice length = 1), constructing a co‐citation reference network containing 2962 nodes and 11902 edges. The network’s modularity *Q* value is 0.76, the weighted average silhouette coefficient *S* value is 0.9074, and the harmonic mean of *Q* and *S* is 0.8272.

Clustering labels of the co‐citation network include triglyceride‐glucose index, gestational weight gain, IADPSG criteria, maternal‐fetal outcome, blood glucose monitoring, gestational impaired glucose tolerance, clinical decision‐making, adiponectin, obstructive sleep apnea, and 75g‐OGTT (Figure 5A,B). The color of each cluster represents temporal changes, and the circle size represents the research frequency of the theme in a specific time period.

Figure 5CiteSpace‐based co‐cited reference cluster view (A) and timeline view (B).

**Figure:**
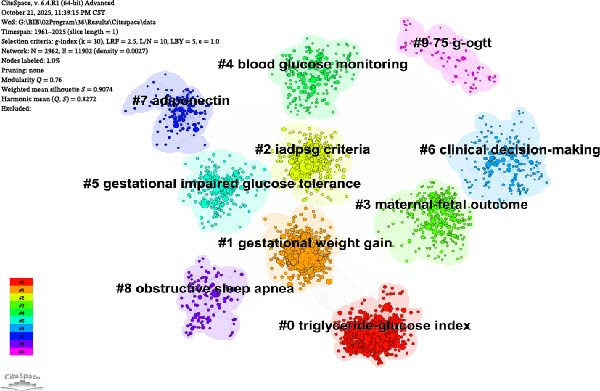
(A)

**Figure:**
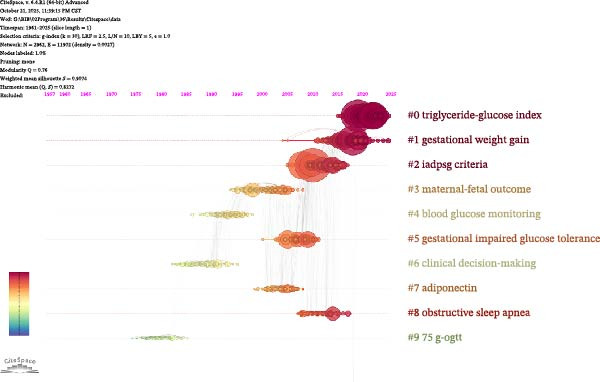
(B)

Table 2 presents the historical evolution of reference literature. Using the Biblioshiny Historiograph analysis tool, we identified literature with significant academic influence by assessing the local citation score (LCS), global citation score (GCS), and total citation counts of each piece of literature.

**Table 2:** Biblioshiny historiograph.

Ranking	Title	Journal	LCS	GCS	Citations	DOI	Publication year
1	Criteria for screening‐tests for gestational diabetes	Am J Obstet Gynecol	649	1581	1726	10.1016/0002–9378(82)90349–0	1982
2	Frequency of gestational diabetes mellitus at collaborating centers based on Iadpsg consensus panel‐recommended criteria the Hyperglycemia and adverse pregnancy outcome (Hapo) study	Diabetes Care	218	565	636	10.2337/dc11‐1641	2012
3	Gestational diabetes mellitus	J Clin Invest	198	750	917	10.1172/JCI200524531	2005
4	Gestational diabetes: The consequences of not treating	Am J Obstet Gynecol	171	494	563	10.1016/j.ajog.2004.11.039	2005
5	Gestational diabetes mellitus and macrosomia: a literature review	Ann Nutr Metab	163	662	806	10.1159/000371628	2015
6	A prospective study of pregravid determinants of gestational diabetes mellitus	Jama‐J Am Med Assoc	152	591	654	10.1001/jama.278.13.1078	1997
7	Impact of increasing carbohydrate intolerance on maternal‐fetal outcomes in 3637 women without gestational diabetes ‐ the toronto tri‐hospital gestational diabetes project	Am J Obstet Gynecol	148	399	448	10.1016/0002‐9378(95)90183‐3	1995
8	First‐trimester fasting Hyperglycemia and adverse pregnancy outcomes	Diabetes care	120	224	253	10.2337/dc09‐0688	2009
9	Gestational diabetes And adverse perinatal outcomes from 716,152 births in France in 2012	Diabetologia	116	389	455	10.1007/s00125–017–4206–6	2017
10	Atlantic diabetes In pregnancy (Dip): The prevalence and outcomes of gestational diabetes mellitus using new diagnostic criteria	Diabetologia	112	239	255	10.1007/s00125–011–2150–4	2011

Abbreviations: GCS, global citation score; LCS, local citation score.

### 3.5. Topic Modeling

We used the LDA algorithm to model topics in the articles, identifying 16 optimal topics according to pairwise cosine distance, Kullback‐Leibler divergence, and model coherence criteria. As shown in Figure 6A,B, these topics are divided into four groups with clear thematic boundaries: the first group is Screening and Diagnostic Approaches, which includes topics #3, #4, #7, and #14, the second group focuses on Pathophysiology and Molecular Mechanisms, which includes topics #5, #8, #9, #15, and #16, the third group is Environmental, Social and Behavioral Determinants, which includes topics #2, #10, and #12 and the fourth group is Clinical Management, Complications and Health Outcomes, which includes topics #1, #6, #11, and #13.

Figure 6Topic modeling fishbone diagram and word cloud. (A) Topic modeling word cloud and (B) topic modeling fishbone diagram.

**Figure:**
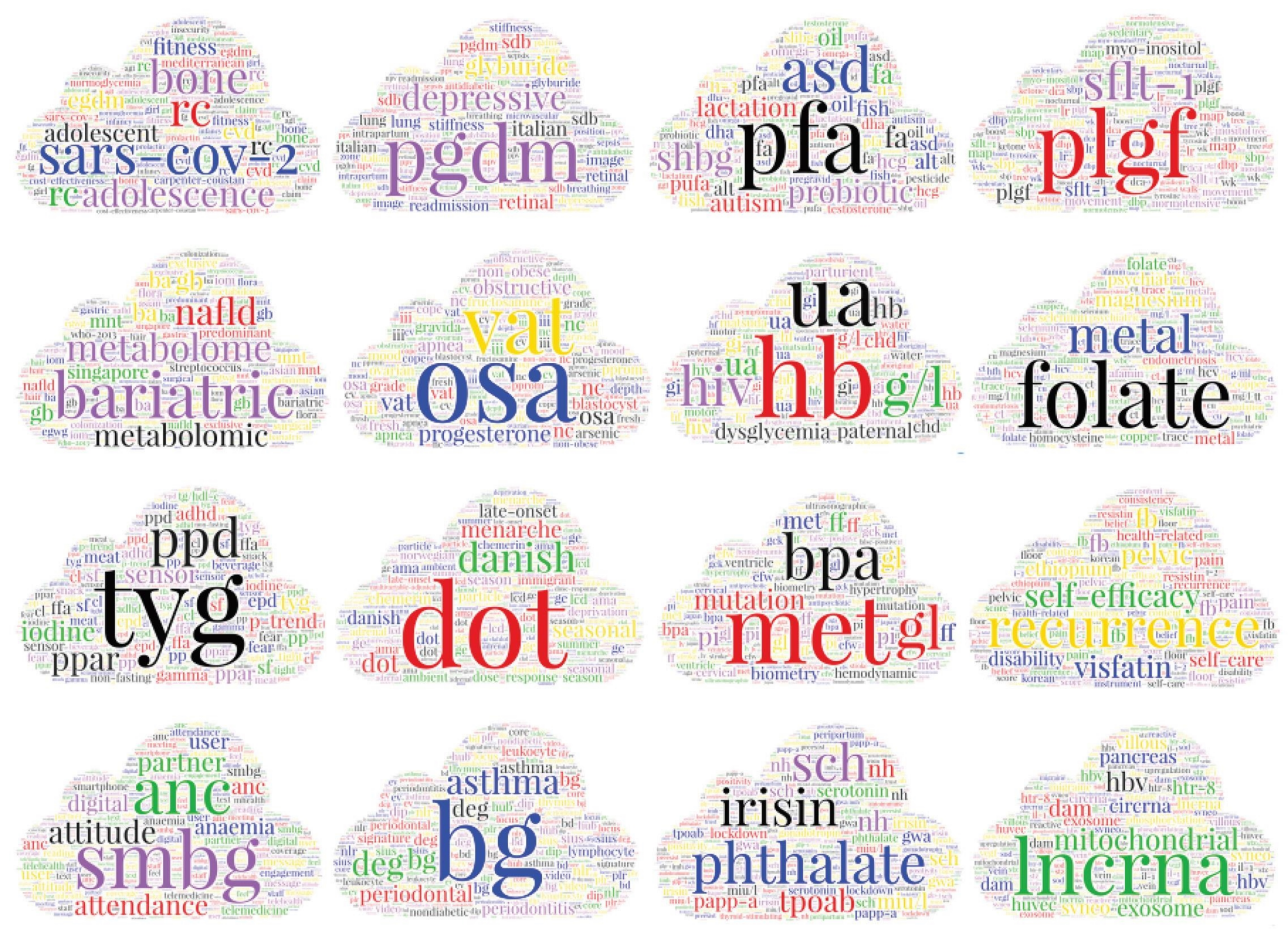
(A)

**Figure:**
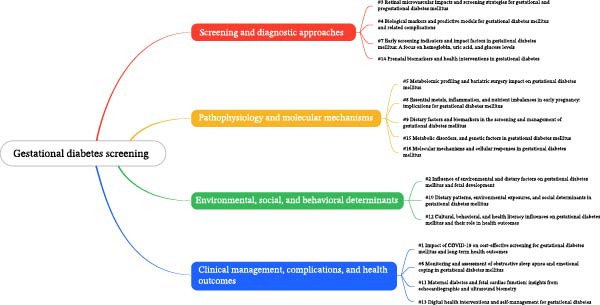
(B)

## 4. Discussion

GDM is a common metabolic disorder during pregnancy, affecting ~14% of pregnancies worldwide. This proportion has been rising alongside obesity and type 2 diabetes, and its incidence has been on a yearly upward trend globally, becoming a major public health concern [24, 25]. Therefore, early identification and management are particularly important. This study systematically analyzed the literature on the GDM screening‐assessment‐monitoring continuum using bibliometric methods integrated with LDA topic modeling, and the discussion will revolve around the empirical findings, combined with clinical and theoretical frameworks to interpret the evolutionary trends and knowledge structure of this field, as well as the study’s incremental contributions and limitations.

### 4.1. Interpretation of Core Empirical Findings

#### 4.1.1. Publication and Collaboration Network Characteristics

Regarding the results of statistical analysis, bibliometric analysis indicates a significant increase in research on gestational diabetes, particularly a 50% increase in the past 5 years. This trend reflects an increased academic focus in this field, partly driven by the global diabetes epidemic and its effects on maternal and infant health. Globally, research on GDM screening shows a clear national distribution pattern, mainly concentrated in economically developed countries and some developing countries. According to the current literature review, economically developed countries such as the United States, Canada, Australia, and some European countries have higher research output and influence. For example, Australia has seen a significant rise in the incidence of gestational diabetes in recent years, with related research focusing on the impact of demographic changes and adjustments in diagnostic standards on prevalence [26]. Additionally, studies in Canada and the United States have revealed differences in GDM prevalence and screening methods in the region through systematic reviews and meta‐analyses [27]. China’s literature output in the field of GDM has shown a significant growth trend, with publication peaks often closely tied to national policies and significant public health events. For instance, after the comprehensive implementation of the two‐child policy in China in 2015, the structure of the pregnant population and pregnancy risks doubled, resulting in a surge in demand for GDM research and a marked increase in related publications. Furthermore, after 2017, with updates to diabetes diagnosis and treatment guidelines and the strengthening of pregnancy health management policies, research interest further intensified. These policy‐driven effects have significantly accelerated the rapid development of GDM research and promoted interdisciplinary collaboration, fostering innovation in screening technologies and intervention strategies [8, 28]. Although research in China has surged in quantity, the average citation per paper is relatively low, suggesting that future research should focus more on “quality over quantity” and participate more in the formulation of internationally influential guidelines (such as HAPO studies).

The collaboration network characteristics show that top research institutions (e.g., University of Toronto) and journals (e.g., Diabetes Care) form tight collaborative clusters, which is conducive to the rapid dissemination of cutting‐edge research results. The high collaboration intensity between obstetrics and gynecology journals and diabetes specialty journals also reflects the interdisciplinary nature of GDM research, which integrates obstetrics, endocrinology, and clinical laboratory medicine.

#### 4.1.2. Keyword and Topic Modeling Evolution: From Diagnosis to Prediction and Monitoring

The three keyword clusters identified in this study correspond to the core links of the GDM clinical management chain: risk factor identification, screening and diagnosis, and pathophysiological mechanism exploration. The LDA topic modeling further divides the field into four thematic groups, clarifying the fine‐grained knowledge structure of the GDM screening‐assessment‐monitoring continuum. The temporal distribution of keywords and citation burst evolution reveals a clear directional shift in the field: the long‐term dominance of OGTT as a core keyword (1990s–2020s) is rooted in methodological path dependance and clinical inertia [29]. Methodologically, OGTT became the de facto gold standard after its inclusion in IADPSG 2010 guidelines and large‐scale epidemiological studies, creating a self‐reinforcing cycle where subsequent research adopted OGTT for comparability, and journals prioritized studies using this standard [30]. Clinically, inertia stems from clinician familiarity with OGTT protocols, limited access to alternative tools in primary care, and concerns about the reproducibility of novel screening methods—these factors have slowed the adoption of promising biomarkers even with comparable diagnostic accuracy [7]. Many studies on biomarkers have focused on metabolites, inflammatory factors, glycosylated proteins, and plasma lipid profiles [31–33]. For example, plasma glycosylated CD59 (pGCD59) shows high diagnostic value in early pregnancy and is significantly associated with GDM and the risk of macrosomia [34]; circulating exosomal piRNAs and circRNAs also show potential as early diagnostic markers for GDM [35]. Additionally, adiponectin and leptin, as indicators regulating glucose and lipid metabolism and inflammatory responses, have levels that are closely related to GDM, with abnormal expressions of leptin and adiponectin potentially indicating aggravated insulin resistance [36]. These biomarkers are characterized by non‐invasive detection, with advantages such as easy collection and high patient acceptance. In summary, research on maternal metabolic and inflammatory indicators provides new perspectives and tools for early screening and management of gestational diabetes, and the construction of multi‐indicator comprehensive assessment models will be an important development direction in this field.

The “two rises” of machine learning and adverse pregnancy outcomes in the citation burst analysis mark a new phase of the field’s development. Unlike the descriptive identification of AI as a hotspot in previous studies, this study clarifies three distinct functional roles of AI/ML in the GDM screening‐assessment‐monitoring continuum: Prediction: ML models integrate clinical characteristics, metabolomics, and genetic data to construct early GDM risk prediction models, addressing the limitation of OGTT’s late diagnosis (24–28 weeks of gestation) [37]. Decision support: AI algorithms analyze real‐world data to optimize screening strategies (e.g., identifying high‐risk subgroups for early screening), improving the cost‐effectiveness of clinical practice [9]. Monitoring: AI‐powered CGM systems provide real‐time glucose trend analysis and alerts, enabling personalized lifestyle interventions and reducing fetal exposure to hyperglycemia [38].

These differentiated roles transform GDM research from a primarily descriptive field to a predictive and interventional one, aligning with the learning health systems theoretical framework where data analytics drive continuous clinical improvement [39].

#### 4.1.3. Environmental, Social, and Behavioral Determinants: A New Dimension of GDM Assessment

The LDA topic modeling identifies Environmental, Social and Behavioral Determinants as an independent core group, which is a key empirical finding of this study. Environmental factors (e.g., pollutants), dietary patterns (e.g., high‐sugar and high‐fat diets), and health literacy affect GDM risk by regulating maternal metabolism, gut microbiota, and inflammatory responses [33]. Unlike traditional research that focuses on individual dietary components, recent studies emphasize the overall effect of dietary patterns—this finding indicates that early pregnancy dietary interventions may be a potential strategy for glucose metabolism regulation. Modern GDM assessment is therefore developing from single blood glucose monitoring to a multidimensional comprehensive model that integrates environmental exposure, psychosocial stress, and behavioral factors, laying the foundation for precision medicine in GDM management.

### 4.2. Incremental Contributions of the Study

The novelty of this study is not merely quantitative bibliometric analysis, but the targeted integration of methods and focus that fills the knowledge gap in existing GDM bibliometric research, with three key incremental contributions: Methodological integration: Unlike prior GDM bibliometric studies that relied solely on citation or keyword analysis, this study combines bibliometric mapping with LDA topic modeling to achieve a dual perspective—identifying “who is publishing what” (collaborative networks) and “what is being studied” (thematic content). This integration uncovers hidden linkages (e.g., North American institutions leading AI prediction research, Asian institutions focusing on CGM‐based monitoring) that single‐method analysis cannot reveal. Focused scope on the screening‐assessment‐monitoring continuum: Existing bibliometric studies of GDM take a broad approach covering pathophysiology, complications, and treatment^8^. By narrowing the focus to this core clinical continuum, this study provides actionable insights for frontline clinicians and policymakers, rather than general field‐wide trends—for example, the differentiation of AI/ML functions directly informs where technological investments can most effectively improve clinical outcomes. Theoretical contextualization of bibliometric patterns: This study links empirical trends to clinical and theoretical frameworks (methodological path dependance, clinical inertia, learning health systems), explaining why the field has evolved in its current direction rather than merely describing the trends. This contextualization elevates the study from pure quantitative analysis to a critical interpretation of the knowledge structure of GDM research.

### 4.3. Limitations of the Study and the Impact of Data Source Selection

Database‐related selection bias is a key potential limitation of this study due to the exclusive use of WoSCC as the data source. Compared with Scopus, WoSCC has insufficient coverage of regional journals focusing on GDM clinical practice in developing countries, which leads to incomplete coverage of screening strategies in specific regions and may underestimate the research progress and practical exploration of GDM screening and monitoring in these areas. In addition, journals indexed by PubMed but not included in WoS (such as some obstetrics and gynecology specialty journals) often publish more clinical guidelines and practice‐oriented research. The lack of these literatures may make the trend analysis of this study overemphasize basic research (such as biomarkers, molecular mechanisms) and relatively ignore clinical implementation research (such as the promotion and optimization of screening guidelines, the exploration of regional personalized screening programs). Although WoSCC has the above‐mentioned coverage limitations, in view of the high overlap rate of WoSCC with other databases in core literatures of the GDM field, and the consistency in the identification of high‐impact research trends, the main research hotspots and long‐term development trends identified in this study still have high reliability and representativeness, and can reflect the mainstream development direction of the global GDM screening, assessment, and monitoring field.

This study has several other limitations in addition to the database selection bias: bibliometric analysis alone cannot provide empirical validation of the effectiveness of screening/monitoring methods, and sample diversity across studies may introduce bias in the generalizability of the results. The study also lacks clinical validation data, limiting the direct applicability of the findings to real‐world clinical settings.

### 4.4. Clinical Implications and Future Research Directions

The empirical findings of this study have clear clinical implications: the shift from OGTT‐based diagnosis to biomarker‐based early prediction and AI‐driven digital monitoring requires the integration of emerging technologies with clinical practice. CGM, as a cutting‐edge monitoring tool, can make up for the limitations of traditional SMBG (e.g., limited measurement frequency, inability to capture glucose fluctuations), and its combination with AI can achieve real‐time dynamic management of GDM patients. Additionally, the identification of environmental and social behavioral factors as core research themes suggests that GDM management should move beyond medical intervention to a comprehensive model that integrates dietary guidance, health education, and social support.

Future research in this field should focus on three core directions: Standardization: Promote international and regional collaboration to establish screening standards adapted to different populations, improving the consistency and generalizability of diagnosis; Technology integration: Strengthen the combination of AI, big data, and traditional screening methods to build intelligent and convenient monitoring platforms for real‐time dynamic management; Individualized management: Conduct multi‐center longitudinal studies to explore optimal screening and intervention strategies for different populations (e.g., different ethnicities and age groups), promoting the implementation of precision medicine in GDM.

## 5. Conclusion

The focus of GDM screening, assessment, and monitoring research is shifting from traditional OGTT‐based diagnosis to biomarker‐based early prediction and AI‐driven digital monitoring throughout pregnancy, with patient characteristics, risk factors, and environmental/social/behavioral determinants emerging as key research focuses. This study systematically analyzes the evolutionary trends and knowledge structure of the GDM screening‐assessment‐monitoring continuum using bibliometric methods integrated with LDA topic modeling, with its incremental contributions lying in the methodological integration, focused clinical scope, and theoretical contextualization of empirical patterns. The findings provide targeted empirical evidence and strategic perspectives for unifying clinical practice, optimizing resource allocation, and guiding future technological innovation in GDM management, ultimately contributing to the improvement of clinical practices and the enhancement of maternal and infant health.

**Nomenclature**
AI: Artificial intelligence
CGM: Continuous glucose monitoring
GCT: Glucose challenge test
GDM: Gestational diabetes mellitus
HbA1c: Glycated hemoglobin
LDA: Latent dirichlet allocation
ML: Machine learning
NLP: Natural language processing
OGTT: Oral glucose tolerance test
SMBG: Self‐monitoring of blood glucose
T2DM: Type 2 diabetes mellitus.

## Author Contributions

Guangzhen Fu contributed to data collection, conducted experiments, and drafted the manuscript. Shuang Hu analyzed the data, validation of results and revised the manuscript. Yunhui Qu designed the study, validation of results, and revised the manuscript.

## Funding

No funding was received for this work.

## Ethics Statement

The authors have nothing to report.

## Conflicts of Interest

The authors declare no conflicts of interest.

## Supporting Information

Additional supporting information can be found online in the Supporting Information section.

## Supporting information


**Supporting Information** Figure S1: Trend of metrics for different methods with the number of topics.

## Data Availability

The data that support the findings of this study are available in Web of Science Core Collection at https://www.webofscience.com/. These data were derived from the following resources available in the public domain: https://www.webofscience.com/.
